# Mobility and strength training with and without protein supplements for pre-frail/frail older people with low protein intake: maximising mobility and strength training (MMoST) feasibility randomised controlled trial

**DOI:** 10.1136/bmjopen-2025-102411

**Published:** 2026-01-07

**Authors:** Esther Williamson, Kavita Biggin, Alana Morris, Ioana R Marian, Christopher Mwema, Andrew Carver, Sarah Elizabeth Lamb

**Affiliations:** 1 Nuffield Department of Orthopaedics, Rheumatology and Musculoskeletal Sciences, University of Oxford, Oxford, UK; 2 Public Health and Sport Sciences, University of Exeter, Exeter, UK; 3 Patient and Public Involvement Representative, University of Oxford, Oxford, UK

**Keywords:** REHABILITATION MEDICINE, NUTRITION & DIETETICS, Frail Elderly, Exercise, Feasibility Studies

## Abstract

**Objectives:**

The first objective was to establish the feasibility of conducting a definitive trial to evaluate the effectiveness of mobility and strength training with or without protein supplements for pre-frail/frail older people with low protein intake. The second objective was to finalise outcome measures for a definitive trial.

**Design:**

Multicentre feasibility randomised controlled trial.

**Setting and participants:**

Four National Health Service (NHS) community trust physiotherapy departments. We recruited via clinical caseloads, an existing cohort study and community advertising. Participants were adults aged ≥60 years, frail or pre-frail, reporting walking difficulties or slow walking and low protein intake (<1 g protein/kg of body weight (kgBW)/day). The recruitment target was 50 participants.

**Interventions:**

All participants undertook two times a week mobility and strength training supported by a physiotherapist for 24 weeks. Half of the participants were randomised (1:1) to receive 24 weeks of daily protein supplements to increase protein intake up to 1.6 g/kgBW/day.

**Primary feasibility objectives:**

Feasibility outcomes assessed recruitment, intervention fidelity, adherence, tolerance and study retention.

**Secondary objectives:**

We assessed clinical data collection at baseline and 5–8 month follow-up including the short physical performance battery (SPPB), 6 min walk test (6MWT) and participant-reported outcomes. Outcome assessors were blinded.

**Statistical methods:**

All participants were analysed in the groups as randomised provided they were not withdrawn from the study before their treatment started and contributed outcome data (modified intention to treat). Our primary feasibility and secondary outcome measures were summarised using descriptive statistics such as mean and SD, median and IQR or counts with percentages. Secondary objectives were exploratory, and mean between group differences at follow-up were estimated for each continuous outcome using linear regression models adjusted for baseline outcome score and frailty status, and presented with associated 95% CIs.

**Results:**

Initially, recruitment focused on existing caseloads, but patients were more unwell and disabled than anticipated and ineligible. No participants were recruited from the cohort. A community recruitment strategy was implemented. We screened 952 older adults and 20 participants were randomised. We ran out of time to reach our target.

We achieved good intervention fidelity for both interventions. The median number of exercise sessions completed was 10.5/16 (IQR 7–13). Six participants received supplements which they tolerated well and took regularly. 14 participants (70%) attended follow-up assessments with no difference in retention between arms.

The median age of participants was 76 years (IQR 68.5–80.0) and 15/20 (75%) were frail. All clinical outcomes showed a trend towards larger improvements in the exercise and protein arm, but these were not statistically significant. For example, SPPB scores (mean difference 0.93, 95% CI (−2.70 to 4.56)) and 6MWT (mean difference 41.92 m, 95% CI (−39.05 to 122.89)) were both higher in the exercise and protein arm compared to control.

**Conclusion:**

The study was not feasible based on the original protocol. Recruitment was the biggest challenge. We established a more efficient route to recruitment (community advertising) which requires further refinement. Clinical outcomes consistently favoured the exercise and protein group, which should be interpreted cautiously but suggest this question is worthy of further investigation.

**Trial registration number:**

ISRCTN30405954.

**STRENGTHS AND LIMITATIONS OF THIS STUDY**

- Outcome assessors were blinded but, as this was a pragmatic study and we did not use a placebo protein supplement, participants could not be blinded to their treatment allocation.
- Recruitment did not proceed as anticipated, and changes were made to the protocol which took time to implement, leaving insufficient time for recruitment.
- Including participants with low protein intake added a level of complexity to this study, but it was an important inclusion criterion as international guidelines recommend the use of protein supplements for older people when dietary intake is insufficient.
- As it was a feasibility study, we cannot draw any conclusions about the effectiveness of the interventions.

## Introduction

 Frailty is a state of vulnerability to adverse outcomes often experienced by older people.^1^ Clinically, older people present with reduced muscle strength and mass and decline in walking ability such as slower walking speed.^2^ These changes can potentially be targeted through rehabilitation. Mobility decline results from changes in muscle mass and strength due to ageing known as sarcopenia. These changes will be further exacerbated by inactivity which is common as people get older. Insufficient dietary protein also contributes to reduced muscle mass and subsequent mobility decline.^3^ Older adults should consume 1–1.2 g protein/kg body weight (BW)/day to maintain muscle mass and function^4^ with higher protein intakes of 1.2–1.5 g/kgBW/day recommended for people living with chronic health problems.^4^ Around 50% of older adults do not consume 1.0 g protein/kgBW/day.^5^ Increasing protein intake using protein supplements is one possible solution for this problem, but there is uncertainty whether this approach is effective in improving muscle strength and mass and slowing or reversing mobility decline. Previous randomised trials have variable results but also differ in terms of setting, patient populations and the dose of protein supplementation.^6^ Research to date suggests that protein supplementation alone is not effective.^7^,^9^ International guidelines recommend that protein supplements should be used when dietary protein is insufficient and provided alongside exercise, but this guidance is based on low and very low certainty of evidence.^6^ Shortcomings of previous trials are short-term follow-up only, including participants who already have sufficient protein intake and failing to individually tailor the dose of protein. High-quality trials with long-term follow-up and economic evaluations are needed.^10^

Exercise including progressive resistance and balance training is recommended to address reduced muscle strength and mass, and mobility decline associated with frailty.^10 11^ We developed and tested a rehabilitation programme for older people that focuses on muscle strength and mobility training,^12^ which resulted in clinically meaningful increases in physical performance including the short physical performance battery (SPPB) and 6 min walk test (6MWT).^13^ Potentially, these benefits could be enhanced by optimising protein intake. We plan to undertake a randomised controlled trial (RCT) to test this hypothesis. However, uncertainty existed about the delivery of such a trial, so we first undertook a feasibility trial to evaluate if the proposed trial could be successfully delivered.

## Objectives

1. To establish if it is feasible to conduct an RCT to evaluate the clinical effectiveness of mobility and strength training with and without protein supplements in pre-frail or frail older adults with low protein intake.
2. To finalise outcome measures for the main RCT by testing data collection procedures and data completeness and to inform the sample size calculations for a main trial.

## Methods

A detailed description of the study protocol including a description of the interventions has been published previously^14^ but we provide details of the study methods below.

### Design

**Figure 1:**
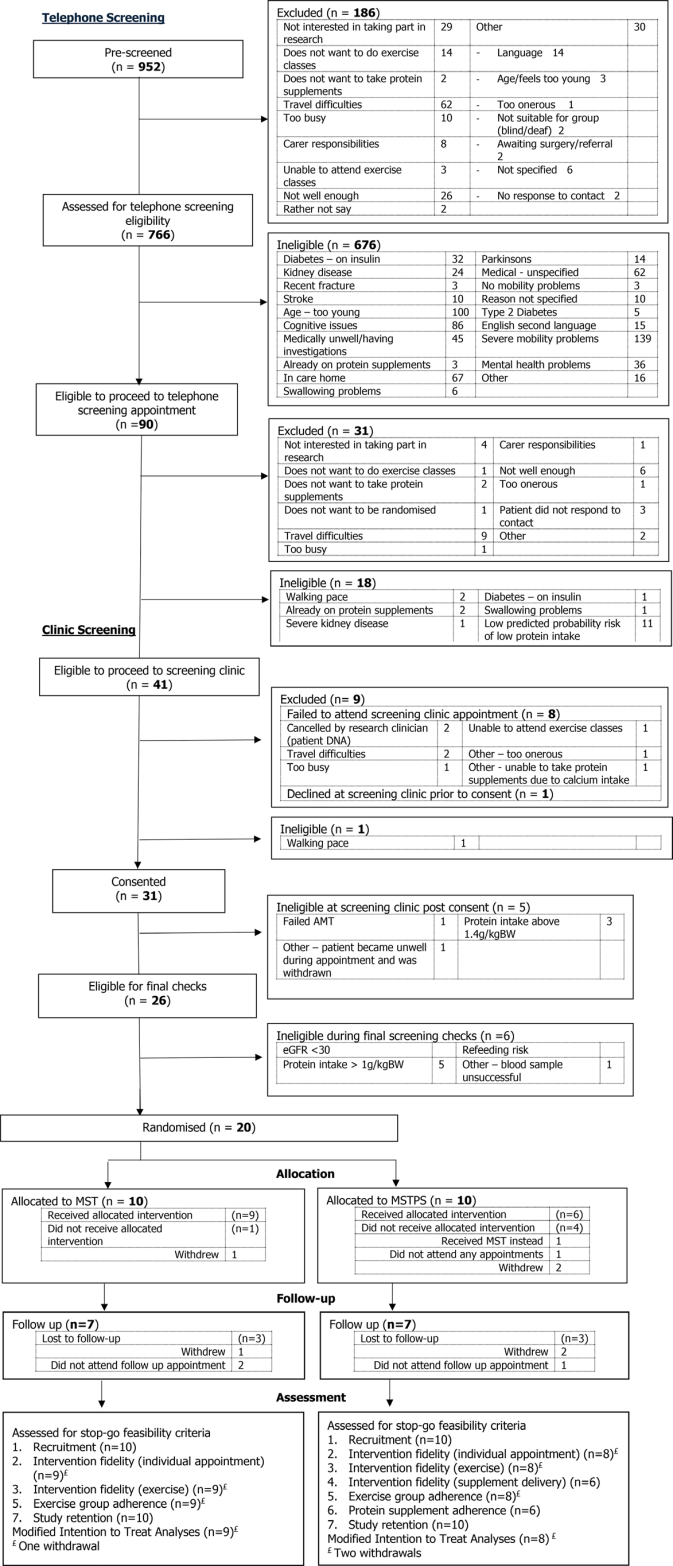
CONSORT diagram. AMT, Abbreviated Mental Test; CONSORT, Consolidated Standards of Reporting Trials; eGFR, estimated glomerular filtration rate; MST, mobility and strength training; MSTPS, mobility and strength training plus protein supplements.

A multicentre, parallel, two-group, feasibility RCT, with (1:1 randomisation) (see figure 1). The study has been reported according to the Consolidated Standards of Reporting Trials (CONSORT) guidance for pilot and feasibility studies.^15^

### Setting

We identified potential participants via:

1. Four National Health Service (NHS) community trusts in England.
2. An existing primary care-based cohort study.
3. Community advertising (introduced in the later phase of recruitment).

Interventions were delivered by community physiotherapy services at the NHS community trusts supported by the study dietitian.

### Study participants and recruitment

Study participants were adults aged ≥60 years who were frail or pre-frail according to the Fried Frailty Criteria,^16^ reported walking difficulties/slow walking and had low protein intake (<1 g protein/kgBW/day). See box 1 for full eligibility criteria. Exclusion criteria were predominantly factors that precluded participants from taking part in group exercise sessions or their suitability for protein supplements.

Box 1 **Eligibility criteria**

**Inclusion criteria**

- Aged 60 years and older.
- Frail (at least three criteria) or pre-frail (1–2 criteria) as defined by the Fried Frailty criteria.^16^ This must include slow walking speed (or difficulty walking).
- Low protein intake (<1 g protein/kg body weight/day) measured by the average of two 24-hour dietary recall assessments.
- Willing and able to provide informed consent to participate.

**Exclusion criteria**

- Dementia or cognitive impairment (defined as an Abbreviated Mental Test score of 6 or less).
- Inability to walk 3 m without assistance (walking aid permitted).
- Unable to follow verbal instructions which would make participation in the exercise group impractical, including severe hearing impairment not corrected by a hearing aid or inability to follow simple safety instructions (eg, English comprehension).
- Living in a residential care or nursing home.
- Pre-existing diagnosis of:
  - Stroke in the last 6 months.
  - Parkinson’s disease.
  - Acute, unstable physical illness that would make participation in the exercise programme unsafe.
  - Dysphagia or swallowing problems that require a modified diet.
  - Type 1 diabetes or Type 2 diabetes on insulin.
  - Severe kidney disease (stage 4 or 5).
- Already taking protein supplements or known allergies to ingredients of protein supplement (milk, soya) or lactose intolerant.
- Poor kidney function defined by an estimated glomerular filtration rate of <30 mL/min/1.73m^2^ (blood test).
- High risk of developing refeeding problems based on items from NICE guidance (https://www.nice.org.uk/guidance/cg32).

NHS community trusts: Clinicians at study sites were asked to identify patients from their caseloads or waiting lists. They gave patients the participant information leaflet (PIL) and asked permission to pass their details to research staff for screening. See online supplemental figure 1 for the participant journey through the study.

Existing cohort study: We had ethical approval to recruit via an existing cohort study (The Oxford Pain, Activity and Lifestyle (OPAL) study).^17^ The characteristics of OPAL cohort participants were broadly similar to the general population of the same age.^17^ Two study sites were suitably located to recruit via the OPAL study. Only one study site was willing to accept OPAL participants. 20 invitation letters and PILs were sent to OPAL participants (based on OPAL questionnaire responses) at this site. They were asked to return a response form to the study team. The other site was unable to accept OPAL participants as they only wanted to recruit from their existing caseload due to waiting list pressures.

Community advertising: Due to poor recruitment, we implemented a new community-based recruitment strategy in the later stages of recruitment. We advertised the study in community venues used by older people, including retirement villages and community centres. Study staff visited venues. Interested people were screened during the visit or they could register to be contacted by the study team on a web form or by phone.

#### Telephone screening

After potential participants were identified, they underwent telephone screening, assessing based on self-report (box 1), and protein intake using a short dietary questionnaire (https://proteinscreener.nl)^18^ to assess if protein intake was likely to be low (defined as ≥30% risk of low protein intake (<1 g/kgBW/day)) and whether they were suitable for further assessment. We asked for verbal permission to view blood test results taken within the last 3 months to check renal function. If eligible, potential participants were invited to attend a research clinic at their local site.

#### Research clinic

Participants attended a research clinic and, if they wished to take part, gave written informed consent and underwent final screening. Following consent, cognitive screening using the Abbreviated Mental Test^19^ and assessment of protein intake were completed. If the participant had not had a blood test in the last 3 months, a capillary blood sample was taken to test estimated glomerular filtration rate (eGFR) using test kits supplied by Medichecks.com Ltd.

Estimated daily protein intake was assessed by taking the average of two 24-hour dietary assessments using an online dietary assessment tool (myfood24.org).^20^ The first dietary analysis was completed during the appointment. Participants were excluded if intake was >1.4 g protein/kgBW/day as they would be unlikely to have an average intake of <1.0 g protein/kgBW/day. The second assessment was completed later by the central study team dietitian via telephone.

The participant was asked to complete a baseline questionnaire and undergo physical tests (table 1).

**Table 1:** Clinic outcomes collected at baseline and follow-up (unless indicated)

Domain	Measure
Demographic	Date of birth, sex, ethnicity, relationship status, index of deprivation (postcode), type of housing, work status, education, carer needs, household income (baseline only) Height and weight (body mass index)
Physical capacity	Short physical performance battery (SPPB)^43^ (proposed primary outcome)
Mobility	6-min walk test^44^
Muscle strength and sarcopenia	Hand grip^45^ and quadriceps strength^46^ measured using dynamometer SARC-F Questionnaire^47^
Frailty	Tilburg Frailty Indicator (TFI)^48^ Clinical Frailty Scale Health Questionnaire to calculate the Clinical Frailty Scale Score^49^ Fried Frailty Index based on self-report^50^
Falls	Prevention of Falls Network Europe (ProFANE) self-report of falls and injuries^51^
Comorbidities	Nordic pain questionnaire^52^ (30, 31); self-reported health conditions^53^
Protein intake	Assessment of daily dietary protein intake (myfood24.org)
Renal function	Blood test to evaluate estimated glomerular filtration rate (eGFR)
Quality of life	EuroQol Group 5-Dimension Questionnaire (EQ-5D-5L)^54^*

* Used with permission.

#### Confirmation of eligibility assessment

The central study team undertook the final eligibility checks including the blood test results and the second dietary assessment prior to randomisation.

### Randomisation

Participants were randomised to exercise (mobility and strength training) or exercise with protein supplements using a secure (encrypted) centralised web-based randomisation service provided by the Oxford Clinical Trials Research Unit. Randomisation was 1:1, stratified by frailty (pre-frail/frail) using variable block sizes of 2 and 4 in a 1:1 ratio. A decision was made to allocate people in the same household to the same treatment based on the participant randomised first due to the risk of contamination.

### Blinding

Following randomisation, the physiotherapy team and the central study team were notified of allocation via automated email. Trial statisticians had access to treatment assignment during the study for the purposes of monitoring randomisation, but data cleaning was done blinded to the allocation. It was not possible to blind the participants or physiotherapists delivering the interventions. Outcome assessors were blinded.

### Interventions

The schedule of intervention delivery is in table 2. More information about the intervention has been published elsewhere.^14^

**Table 2:** Intervention delivery schedule

	Mobility and strength training	Training + protein supplements
*Individual pre-class appointment*		
Assessment by physiotherapist to establish current ability and exercise setting. Home exercise diary introduced.	x	x
Assessment by physiotherapist to discuss protein supplement dosing and instructions. Provided with written instructions and introduced to protein supplement diary.		x
Delivery of protein supplement arranged.		x
*Weeks 1–16*		
Weekly group mobility and strength exercises Group discussion including behavioural strategies to encourage adherence with home exercises (goal setting, exercise planning, exercise diary completion and problem solving) (15 min). Seated warm up (5 min). Arm raisesx10. Trunk rotationsx10. Knee liftsx10. Pelvic tiltingx10 Exercise circuit (individually tailored; 20 min)*: Strengthening exercises: knee extension, hip extension and abduction, sit to stand. Combined hip flexor and calf stretch. Balance exercise. Walking circuit (20 min) One session of home exercises each week (exercise diary completed). Weights for home exercises were provided if appropriate.	x	x
Protein supplements taken daily (individually tailored based on dietary assessment). Up to two servings of Fortifit (powered supplement) to provide up to 1.6 g protein/kgBW/day. Complete supplement diary.		x
Protein supplement reviews including weight checks undertaken at weeks 2, 4, 8, 12 and 16 of the programme^†^. Referral to dietitian if needed.		x
*Weeks 17–24*		
Two times a week home exercises (exercises as described above). Complete exercise diary.	x	x
Three follow-up telephone calls to review progress with home exercises conducted by physiotherapist.	x	x
Protein supplements taken daily as described above. Complete the supplement diary.		x
Three telephone supplement reviews during the follow-up telephone calls. Weight is not monitored.		x

* During the strengthening exercises, participants were asked to work at a level of 5–6/10 on the Borg Rating Scale of Perceived Exertion. Load has to be added using weights or resistance bands. Speed was added to introduce a power element.

† All participants attended the same exercise sessions. Supplement reviews were conducted outside these sessions, and participants were asked not to discuss them in the groups.

#### Exercise: mobility and strength training

All participants undertook two times a week exercise supported by a physiotherapist for 24 weeks using a combination of weekly group exercise (16 weeks) and home exercises (8 weeks). The group exercise sessions were held in the physiotherapy departments of the Community NHS Trust. Transport was not provided. Three out of the four sites offered some funding to cover travel costs (local arrangements) but participants had to make their own way to the exercise groups. The programme was progressive and tailored to each participant. Participants attended an individual appointment with the physiotherapist for baseline setting prior to attending the exercise group. Each group session followed the same format, starting with a group discussion to promote exercise adherence and followed by an exercise circuit and supervised walking circuit. The exercises are listed in table 2. The exercises performed during the group sessions were also undertaken at home, including the walking.

Participants completed the programme of exercises and walking at home once a week while attending the group. These were then completed two times a week during the independent exercise phase (another 8 weeks). Participants received three support phone calls during this time.

#### Protein supplements

Participants randomised to protein supplements were asked to take supplements daily for 24 weeks. The protein was a powdered supplement (Fortifit, Nutricia) delivered to participants’ homes. Each serving consisted of 40 g of powder (vanilla or strawberry flavour), providing 150 kcal and containing 20 g of whey proteins, 2.8 g of leucine, 9 g of carbohydrates, 8 g of fat, 800 IU of vitamin D, a mixture of vitamins, minerals (calcium, 500 mg) and 1.3 g of dietary fibre. The maximum dose was two servings per day due to the vitamin D and calcium content, as additional servings would have exceeded the daily recommended dose limits.

Physiotherapists provided the supplement to participants with the support of the study dietitian. Dosage was tailored based on baseline protein intake. The aim was to increase intake up to 1.6 g protein/kgBW/day^21^ based on a breakpoint analysis identifying when gains due to increased protein plateau.^21^ This is slightly higher than the recommended daily protein intake for older adults with acute or chronic diseases to maintain or regain muscle mass (1.2–1.5 g protein/kgBW/day).^4^ If a participant had a body mass index of ≥30, then their prescribed dose of protein was 75% of the amount based on weight (as per usual clinical practice in the UK).^22^ If a participant had a moderate reduction in renal function measured by their eGFR (between 30 and 60 mL/min/1.73 m^2^), protein intake was reduced to 1.3 g/kgBW/day based on The Kidney Disease Improving Global Outcomes guidelines.^23^

On a day when participants undertook the exercises, they were asked to take one serving directly after the session. Otherwise, participants were instructed to take one serving before breakfast and the second serving (if prescribed) before their lunch or dinner. Participants completed a diary to record intake and side effects. Participants were monitored and weighed at regular reviews by the physiotherapists. The physiotherapists were trained to address straightforward issues with supplement adherence, for example, offering a different flavour, providing a recipe booklet to make the supplement more palatable or to advise the participant to avoid taking the supplement with meals if it was reducing their appetite. If a participant lost >10% of their weight or reported ongoing intolerance issues, then they were reviewed by the study dietitian. Reviews were conducted over the telephone and issues related to weight loss or tolerance were assessed and tailored advice provided. Regular routines were continued by the physiotherapist, and they were referred back to the study dietitian if required.

### Outcome measures

#### Primary feasibility success criteria

The assessment of study feasibility was based on the stop-go criteria in table 3 which used a ‘traffic light’ system: red (stop), amber (proceed with modifications) and green (go).^24^ We took a pragmatic approach to the stop-go criteria. They were decided by the study team based on published recommendations,^24 25^ our experience of delivering large clinical trials and our understanding of exercise and protein supplement dosing and through discussion with our patient and public involvement (PPI) group.

**Table 3:** Stop-go criteria

	Red	Amber	Green	Study findings
Recruitment: number of participants recruited*	<25 participants recruited within 6 months	25–39 participants recruited within 6 months	>40 participants recruited within 6 months	**20/50 participants**
Intervention fidelity: provision of the mobility and strength training	<50% undergo their individual assessment and are allocated to their group sessions	50%–80% of participants undergo their individual assessment and are allocated to their group sessions	>80% of participants undergo their individual assessment and are allocated to their group sessions	**80** **%** **attended their individual appointment**
Intervention fidelity: delivery of the mobility and strength training (fidelity assessment via observations based on key criteria checklist)	<50% of all key criteria are met during observed sessions	50%–80% of key criteria are met during observed sessions	>80% of key criteria are met during observed sessions	**95** **%** **of key criteria met**
Intervention fidelity: protein supplements	<50% of participants receive the protein supplements via home delivery as intended	50%–80% of participants receive the protein supplements via home delivery as intended	>80% of participants receive the protein supplements via home delivery as intended	**>** **80%** **received the protein** **supplements via** **home delivery**
Intervention adherence: participant attendance at the mobility and strength training	On average, <8/16 sessions (50%) of sessions attended by 70% or more participants	On average, 8–10/16 sessions (50%–69%) of sessions attended by 70% or more participants	On average, at least 11/16 sessions (70%) attended by 70% or more of participants	**70.6** **%** **participants attended at least** **eight** **sessions**
Intervention adherence: participant adherence to the protein supplements	On average, 70% or more participants report taking protein supplements on <50% of days over 24 weeks	On average, 70% or more participants report taking protein supplements on 50%–70% of days over 24 weeks	On average, 70% or more participants report taking protein supplements on ≥70% of days over 24 weeks	**100** **%** **of participants who received the supplements took them on more than** **70** **%** **of days**
Study retention: follow-up rates and checking for differential loss to follow-up	>30% loss to follow in either study arm.	30%–15% loss to follow-up in either study arm.	<15% loss to follow-up in both study arms.	**30** **%** **attended** **follow-up** **No differential loss to** **follow-up**

* To achieve the recruitment target of 50, the aim was to recruit approximately two participants/month/site over a 6-month recruitment period at the four sites.

##### Criteria 1 (recruitment)

Green light was set at 40 participants over 6 months which was 80% of the recruitment target, as we expected recruitment to be challenging and the potential need for adaptations over the course of the study.

##### Criteria 2 (intervention fidelity: delivery provision of mobility and strength training)

Green light was set at >80% of participants attending for their individual assessment. This was considered a reasonable target as older adults who are frail often find it difficult to attend appointments. For example, published non-attendance rates by frailer older people for falls prevention services can be as high as 35%.^26 27^

##### Criteria 3 (intervention fidelity: delivery of the mobility and strength training (fidelity assessment via observations based on key criteria checklist))

Green light was set at >80% of key criteria fulfilled as an indicator of high fidelity based on previous research and guidance.^28 29^

##### Criteria 4 (intervention fidelity: protein supplements)

There was uncertainty around how well the home delivery of protein supplements would work and whether they would be delivered in time to start taking them on the first day of the exercise programme. A green light of >80% was set, which was a pragmatic decision.

##### Criteria 5 (intervention adherence: attendance at the mobility and strength training)

To ensure an adequate dose of exercises, green light was set at the majority (70%) of participants attending at least 70% of sessions. This was based on participant attendance at a similar programme demonstrated to be effective in a previous trial conducted by our team.^13^

##### Criteria 6 (intervention adherence: participant adherence to the protein supplements)

Green light was set at the majority (70%) of participants taking their supplements on >70% of days (on average 5 out of 7 days or more each week). Compliance definitions from other protein supplementation trials informed this decision.^30^

##### Criteria 7 (study retention: follow-up rates and checking for differential loss to follow-up)

>20% loss to follow is considered a threat to the validity of a trial.^31^ Green light was set at <15% follow to account for further attrition at 12 months follow-up (planned primary time point for a definitive trial).

### Secondary outcomes

We collected clinical and participant-reported data at baseline and follow-up (5–8 months post randomisation). This was done to (1) determine the viability of data collection (including the time taken to undertake data collection and burden on participants, any assistance needed by participants, if the researcher correctly followed the protocol and the completeness of data) to refine the protocol for a definitive RCT; (2) inform the sample size calculation for a definitive RCT based on the proposed primary outcome (SPPB) (see table 1). The original follow-up time point was 8 months, but this was modified to 5–8 months to ensure all participants completed follow-up before the end of the study.

### Adverse events

Adverse events (AEs) were collected throughout the study by research staff and physiotherapists. All staff were trained to report AEs, and we followed reporting procedures in accordance with Good Clinical Practice.

As study participants were older adults, there were foreseeable AEs that were reported if they occurred during or within 2 hours of any study interventions. These included acute infections, medical instability (eg, worsening of heart failure), vestibular disorders or stroke and fall-related injuries. We expected participants may experience delayed onset muscle soreness (<72 hours) due to starting a new exercise programme, and this was only reported if they were more persistent or severe than expected. Participants taking protein supplements sometimes experience mild gastrointestinal complaints including diarrhoea, constipation, bloating and loss of appetite. If these were more severe and longer lasting than what was expected, then they were reportable.

### Sample size

The target sample size was 50 based on a recent simulation study of sample size requirements in pilot RCTs which recommends at least 50 subjects are needed to calculate key design parameters and establish the definitive study sample size.^32^

### Statistical methods

The analyses were conducted according to a pre-specified statistical analysis plan described in the study protocol. The primary analysis evaluated the feasibility of conducting a definitive RCT. Descriptive statistics of the feasibility outcomes are reported (recruitment rates, reasons for ineligibility, intervention fidelity and adherence, retention rates). Participant baseline demographic and clinical characteristics for each randomised group and overall are described.

Participant-reported outcomes are summarised as a mean (SD) or median (IQR) and frequency with percentages for continuous and categorical outcomes, respectively. Normality was assessed using histograms and boxplots for each continuous outcome. We report medians with IQR to summarise continuous outcomes as the sample size in our feasibility study was small. The mean between group differences at follow-up were estimated for each continuous outcome using linear regression models adjusted for baseline outcome score and frailty status, and presented with associated 95% CIs. These were exploratory analyses to test for a possible signal of treatment effect in the data. No p values were included as the study was not powered for between group comparisons. All participants who were randomised and not withdrawn from the study before their treatment started and provided data were included in the analyses (modified intention to treat).

### Patient and public involvement

We established a PPI group of older people who were involved in the design, conduct and reporting of this study. They helped to develop study materials and test procedures such as the protein assessments. We had PPI representatives on the trial management committee and oversight committee where they took part in discussions about and reviewed the protocol including selection of outcomes and setting stop-go criteria. Throughout the study, the Chief Investigator met with the PPI group to update them on progress, discuss challenges and possible solutions and finally, the results with discussions around the design of a definitive trial.

## Results

The flow and number of participants at each stage is shown in the CONSORT diagram (figure 1). The feasibility outcomes are summarised in table 3.

### Feasibility outcomes

#### Recruitment: stop-go criteria=red

Recruitment opened on 1 March 2023. The first participant was randomised on 10 July 2023. The original recruitment period was 4 months which we extended to 6 months (online supplemental table 1). Scoping of caseloads during set up had indicated there would be more than enough suitable patients. However, when sites screened patient lists, patients were ineligible due to poor health or declined to take part (see figure 1 for reasons for exclusion). In May 2023, we proposed a new route of recruitment via community advertising. Some sites were reluctant to recruit from outside of their caseloads due to waiting list pressures, but this was eventually implemented at all sites during July and August (following REC approval). This was a more efficient route with fewer people being screened to convert to randomised participants. We stopped identifying new potential participants on 30 September 2023. Eligibility assessments were then completed with the last participant randomised on 20 November 2023.

In total, 20 participants were randomised (10 in each arm). We recruited 13 participants via community recruitment (from initial screening of 99 older adults (13% screened)) and 7 via NHS sources (from initial screening of 816 patients (519 from one site (0.8% screened; 6 recruited from one site))). No participants were recruited via the OPAL cohort study (37 OPAL participants invited).

##### Participant identification

In total, 952 older adults were screened to identify potential participants. Of these, 90/952 (9.5%) were potentially eligible and gave permission to pass their contact details to research staff. The most common reasons for ineligibility were severe mobility problems (eg, housebound; n=139/952 (14.6%)) and being too young (n=100/952 (10.5%)). Most screening was done using physiotherapy referrals and medical records. In these cases, if participants appeared eligible from their physiotherapy referral or medical record, then they were telephoned by the clinical staff to seek permission to pass their details to the research staff. Nearly 20% (186/952) did not give permission. The most common reason for declining was travel difficulties (n=62/186 (33%)).

##### Telephone screening

Research staff contacted 87/90 potential participants who underwent telephone screening (three uncontactable), of which 28 (32%) declined to take part. The most common reason for declining was travel difficulties (9/28; 32%). Of those found to be ineligible (18/87 (21%)), the most common reason was they were unlikely to have low protein intake (11/18).

##### Research clinics

Research staff invited 41 patients to research clinic appointments and 33 (81%) attended with 31/33 (94%) consenting to undertake final eligibility assessments and participate in the study. Final eligibility checks excluded 11 participants. The most common exclusion was high dietary protein intake (8/11). 20 participants were randomised.

### Intervention fidelity

#### Stop-go criteria for fidelity assessments of the mobility and strength training sessions=green

Exercise groups were delivered at three sites (sites 1–3). One site (site 4) only recruited two participants, and neither could attend the proposed time/day; therefore, the intervention was delivered via home visits. The exercise groups were observed at the three sites delivering them. They were delivered with a high level of fidelity, with 95% of the core criteria being met. Participants were observed to be engaged with the groups and enjoyed taking part.

#### Stop-go criteria for attendance at individual appointment=amber

80% of participants (16/20) attended their individual appointment where they were told their treatment allocation. Three participants withdrew before attending this appointment (exercise only=1; exercise and protein=2), and one participant failed to attend any sessions (exercise and protein arm). Two withdrawals were health-related, and one was not specified.

#### Stop-go criteria for home delivery of protein supplements=green

Six out of 10 participants randomised to protein supplements received them. Two participants withdrew before attending any intervention appointments and prior to knowing their allocation, and one participant did not attend any intervention sessions (allocation also unknown to participant). One participant did not receive protein supplements as he was participating with his wife who was randomised first to receive exercise only. Therefore, he was given the same treatment (see the section Randomisation).

Of the six participants who attended their first appointment, and a supplement order was made, supplements were delivered as intended >80% of the time. There were some delivery issues over the Christmas period as stock of vanilla flavoured supplements ran out, due to this flavour being preferred by all participants over strawberry.

### Intervention adherence

#### Stop go-criteria for exercise group attendance=amber

Across all four sites, excluding withdrawals, the median number of exercise sessions completed was 10.5 out of a possible 16 (IQR 7–13) with 12/17 participants (70.6%) attending at least 8/16 sessions (amber) (table 4).

**Table 4:** Exercise sessions completed (n=17, excluding withdrawals)

Number of sessions attended*	Number of participants (n=17)
No session attended	1 (5.9)
Two sessions	1 (5.9)
Six sessions†	3 (17.6)
Eight sessions	2 (11.8)
Nine sessions	1 (5.9)
10 sessions	1 (5.9)
11 sessions	2 (11.8)
12 sessions	1 (5.9)
13 sessions	3 (17.6)
14 sessions	1 (5.9)
15 sessions	1 (5.9)

* Summaries are n (%).

† Site 4 participants (n=2) received six home visits to deliver the exercise intervention at weeks 1, 2, 4, 8, 12 and 16.

When data are limited to the three sites delivering group sessions, including withdrawals, 12/18 randomised (66.66%) attended at least 8/16 sessions, falling slightly short of amber stop-go criteria. The main reason for non-attendance was sickness.

#### Stop-go criteria for adherence to the protein supplements=green

Participants were prescribed supplements for 168 days. Among those receiving supplements (n=6), they reported taking the supplements on a median of 140.5 days (83%) (IQR 126–159) and all participants took them on >70% of days. The full prescribed dose was taken on average on 75% of days (partial dose, 8%).

We had aimed to provide participants with a dose of protein of up to 1.6 g/kgBW/day. Examining all participants allocated to exercise and protein, the majority (8/10 (80%)) were prescribed the maximum two drinks per day (40 g protein). The total daily protein intake that would have been achieved if participants took the prescribed supplement is listed in online supplemental table 2). Only 1/10 (10%) would have achieved a protein intake of 1.5–1.6 g/kgBW/day, with 1.4–1.49 g/kgBW/day being the most common dose achieved (3/10 (30%)). Some participants had such large dietary protein gaps that they fell well short of 1.6 g/kgBW/day even with two drinks. 8/10 (80%) participants would have received a dose between 1.2 and 1.6 g/kgBW/day which is recommended for older people with chronic disease.^4^ Participants taking the supplements tolerated them well and did not report any AEs/side-effects.

### Study retention: stop-go criteria=amber

There was no observed differential loss to follow-up. 14/20 (70%) completed the physical tests. 15/20 (75%) completed the patient-reported outcomes (questionnaire).

### Baseline characteristics and clinical outcomes

At baseline, there was a 100% completion rate of all outcomes. There was very little missing data among participants who attended for follow-up. Among participants who attended a follow-up research clinical appointment (n=14), there was a 100% completion rate for all the physical measures. For the follow-up questionnaire (n=15), the completion rate was also 100% for all participant completed questions. One participant who responded at follow-up did not complete the follow-up dietary assessment and there were three missing results for follow-up eGFRs.

The median age of participants was 76 years (IQR 68.5–80.0). Most were frail (15/20 (75%)) and the rest were pre-frail according to the Fried Frailty Criteria used to establish study eligibility. The majority were white (18/20 (90%)) and women (15/20 (75%)) (table 5). There was an imbalance in some baseline characteristics given the number of randomisations. Those in the exercise and protein arm were younger and more physically able than those in the exercise only arm. The majority in both arms had suspected sarcopenia based on the SARC-F questionnaire and were frail based on the Tilburg Frailty Index.

**Table 5:** Participant baseline characteristics (n=20)

	Mobility and strength training (n=10)	Mobility and strength training plus protein supplements (n=10)	Total (n=20)
Frailty level*			
Pre-frail	2 (20)	3 (30)	5 (25)
Frail	8 (80)	7 (70)	15 (75)
Participant age (years)†	79.0 (72.0–81.0)	72.5 (66.0–77.0)	76.0 (68.5–80.0)
Gender*			
Male	1 (10)	4 (40)	5 (25)
Female	9 (90)	6 (60)	15 (75)
Body mass index (BMI), kg/m^2^†	31.9 (26.6–37.0)	31.1 (26.6–44.0)	31.6 (26.6–37.8)
Ethnicity			
White	9 (90)	9 (90)	18 (90)
Mixed	1 (10)	0 (0)	1 (5)
Black or black British	0 (0)	1 (10)	1 (5)
Current relationship status*			
Married/civil union	5 (50)	5 (50)	10 (50)
Unmarried (never married)	2 (20)	2 (20)	4 (20)
Separated/divorced	1 (10)	1 (10)	2 (10)
Widow/widower	2 (20)	2 (20)	4 (20)
Level of education*			
None or primary education	0 (0)	2 (20)	2 (10)
Secondary education	6 (60)	4 (40)	10 (50)
Higher professional or university education	4 (40)	4 (40)	8 (40)
Work status*			
Retired	10 (100)	8 (80)	18 (90)
Semi-retired	0 (0)	1 (10)	1 (5)
Employed	0 (0)	1 (10)	1 (5)
Place of residence*			
Owner occupied house/flat	9 (90)	6 (60)	15 (75)
Privately rented house/flat	1 (10)	1 (10)	2 (10)
Rented from housing association/local authority	0 (0)	3 (30)	3 (15)
Unpaid carer*			
No	8 (80)	7 (70)	15 (75)
Yes	2 (20)	3 (30)	5 (25)
Paid carer*			
No	9 (90)	10 (100)	19 (95)
Yes	1 (10)	0 (0)	1 (5)
Total annual household income*			
£7500 to <£10 000	3 (30)	2 (20)	5 (25)
£10 000 to <£20 000	1 (10)	3 (30)	4 (20)
£20 000 to <£30 000	0 (0)	4 (40)	4 (20)
£30 000 to <£40 000	2 (20)	0 (0)	2 (10)
£40 000 to <£50 000	1 (10)	0 (0)	1 (5)
Prefer not to answer	3 (30)	1 (10)	4 (20)
Total SPPB Score*‡	5.0 (4.0–7.0)	8.0 (7.0–8.0)	7.0 (4.5–8.0)
6 min walk test (m)*§	133.5 (109.8–160.0)	189.4 (161.1–235.0)	160.6 (115.0–218.1)
Hand grip strength; right (kg)*^¶^	18.4 (12.8–19.6)	19.7 (11.7–29.4)	18.9 (12.2–22.5)
Hand grip strength; hand (kg)*^¶^	15.4 (11.9–16.5)	20.9 (11.1–26.5)	15.8 (11.4–22.4)
Participant’s dominant hand†			
Left	1 (10)	1 (10)	2 (10)
Right	9 (90)	9 (90)	18 (90)
Quad strength; right foot*^¶^	17.8 (14.1–22.8)	13.9 (7.1–32.7)	14.8 (10.8–25.8)
Quad strength; left foot*^¶^	14.4 (8.4–18.1)	19.8 (7.8–35.8)	14.7 (8.1–28.4)
SARC-F Questionnaire score*	5.5 (4.0–7.0)	4.5 (2.0–5.0)	5.0 (4.0–6.0)
SARC-F Questionnaire†			
Asymptomatic	1 (10)	3 (30)	4 (20)
Symptomatic	9 (90)	7 (70)	16 (80)
Tilburg Frailty Indicator (TFI)†**			
Not frail	2 (20)	2 (20)	4 (20)
Frail	8 (80)	8 (80)	16 (80)
Clinical Frailty Scale Health Questionnaire*^††^	6.0 (4.0–7.0)	4.5 (3.0–5.0)	5.0 (3.0–6.0)
Estimate of the Fried Frailty Index* ^‡‡^	2.5 (2–4)	2 (2–3)	2 (2–3)
Fall (last 12 months)†			
I have not fallen	2 (20)	5 (50)	7 (35)
I have fallen once	2 (20)	3 (30)	5 (25)
I have fallen more than once	6 (60)	2 (20)	8 (40)
Fractures resulting from falls in the last 12 months†			
No	8 (80)	10 (100)	18 (90)
Yes	2 (20)	0 (0)	2 (10)
If yes, number of broken bones*	1.5 (1.0–2.0)	0 (0–0)	1.5 (1.0–2.0)
Nordic pain questionnaire (multisite)†			
Single-site pain	1 (10)	1 (10)	2 (10)
Multisite musculoskeletal pain	8 (80)	7 (70)	15 (75)
Missing	1 (10)	2 (20)	3 (15)
Self-reported health conditions*			
None	2 (20)	0 (0)	2 (10)
One or more	8 (80)	10 (100)	18 (90)
Daily protein intake measured over 2 days (g)*	0.70 (0.50–0.80)	0.80 (0.60–0.90)	0.70 (0.60–0.80)
EQ-5D Utility Score* ^§§^	0.625 (0.422–0.666)	0.598 (0.527–0.757)	0.617 (0.497–0.738)
EQ-5D VAS* ^¶¶^	67.5 (55.0–75.0)	70.0 (60.0–80.0)	67.5 (57.5–80.0)

* Summaries are median (IQR) or n (%), where n indicates missing data.

† Summaries are n (%) unless stated otherwise.

‡ Short physical performance battery (SPPB) (0–12, lower values worse).

§ 6-min walk test calculates as number of laps times length of lap, plus length of last lap (metres, higher values indicating more metres walked).

¶ Highest value of the three attempts was reported for the hand grip strength and quadriceps strength, with higher scores indicating better strength.

** Tilburg Frailty Index (0–15, higher values worse).

†† Clinical Frailty Scale Health Questionnaire (0–9, higher values indicating worse frailty).

‡‡ Fried Frailty Index (0–5, higher values worse).

§§ EQ-5D Utility Score (−0.594 to 1, higher scores indicating better quality of life and a score of 0 is considered equivalent to death).

¶¶ EQ-5D VAS (0–100, higher scores indicating better health state.

EQ-5D, EuroQol Group 5-Dimension; VAS, Visual Analogue Scale.

Follow-up outcome data showed that after adjusting for baseline values, physical tests and patient completed outcome measures demonstrated a trend towards greater improvements in the exercise and protein group compared with exercise only over the 5–8 months study period (table 6). Although these between-group differences were not statistically significant and they had wide CIs, findings were consistently in favour of the exercise and protein arm. For example, greater improvement in SPPB was observed in the exercise and protein group with a between-group difference of 0.93 (95% CI −2.70 to 4.56). Similarly, there were greater improvements in 6MWT results with a between-group difference of 41.92 (95% CI −39.05 to 122.89).

**Table 6:** Clinical outcomes: between group differences adjusted for baseline values and frailty status

	Mobility and strength training (n=9)		Mobility and strength training plus protein supplements (n=8)		Between-group difference (95% CI)*	Scale, favours exercise/exercise plus protein group
	n	Unadjusted mean (SD)	n	Unadjusted mean (SD)		
Short physical performance battery						
Baseline	9	5.8 (2.3)	8	7.1 (2.4)	–	
5–8 months	7	6.6 (3.7)	7	8.4 (3.3)	0.93 (−2.70 to 4.56)	0–12, Protein
6 min walk test						
Baseline	9	151.1 (73.5)	8	203.2 (91.3)	–	
5–8 Months	7	165.5 (90.8)	7	250.9 (115.5)	41.92 (−39.05 to 122.89)	40–390 m, Protein
Hand grip strength (kg)—right						
Baseline	9	16.8 (5.3)	8	19.4 (10.1)	–	
5–8 Months	7	18.0 (4.5)	7	22.8 (10.4)	3.21 (−1.32 to 7.74)	7.1–34.6 kg, Protein
Hand grip strength (kg)—left						
Baseline	9	14.3 (5.1)	8	18.5 (11.0)	–	
5–8 Months	7	17.0 (2.4)	7	21.6 (10.4)	1.88 (−3.43 to 7.19)	3.0–36.1 kg, Protein
Quadriceps strength (kg)—right						
Baseline	8	18.4 (5.8)	8	23.6 (15.5)	–	
5–8 Months	7	20.1 (6.8)	7	36.9 (21.9)	9.76 (−10.60 to 30.11)	4.3–47.2 kg, Protein
Quadriceps strength (kg)—left						
Baseline	9	18.5 (19.8)	8	27.2 (19.9)	–	
5–8 Months	7	16.6 (7.4)	7	32.8 (9.9)	12.17 (1.60 to 22.74)	2.9–67.5 kg, Protein
SARC-F Questionnaire						
Baseline	9	5.6 (2.2)	8	4.2 (1.6)	–	
5–8 Months	7	5.0 (2.8)	8	2.6 (2.3)	−1.61 (−4.19 to 0.96)	0–10, Protein
Tilburg Frailty Indicator (TFI)						
Baseline	9	6.8 (2.3)	8	5.4 (1.2)	–	
5–8 Months	7	6.6 (2.0)	8	4.9 (2.2)	−1.14 (−3.64 to 1.35)	0–15, Protein
eGFR (mL/min/1.73 m^2^)						
Baseline	9	69.3 (16.3)	8	65.9 (19.0)	–	
5–8 Months	7	69.9 (15.7)	5	57.2 (27.5)	−2.28 (−14.65 to 10.09)	0–120, Exercise only
EQ-5D Utility Score						
Baseline	9	0.5 (0.3)	8	0.6 (0.2)	–	
5–8 Months	7	0.5 (0.4)	8	0.7 (0.2)	0.158 (−0.105 to 0.420)	−0.594 to 1, Protein
EQ-5D VAS						
Baseline	9	62.2 (17.9)	8	67.1 (14.9)	–	
5–8 Months	7	56.4 (22.9)	8	68.8 (18.9)	10.87 (−14.02 to 35.77)	0–100, Protein
Estimate of the Fried Frailty Index						
Baseline	9	2.9 (0.9)	8	2.1 (0.6)	–	
5–8 Months	7	2.9 (0.9)	8	1.9 (1.0)	−0.48 (−1.46 to 0.51)	0–5, Protein
EQ-5D Pain Score						
Baseline	8	3.0 (2.5)	6	4.5 (2.1)	–	
5–8 Months	7	4.4 (3.0)	8	3.6 (2.0)	−0.15 (−1.40 to 1.10)	1–5, Protein
Daily dietary protein intake measured over 2 days (g)						
Baseline	9	0.7 (0.2)	8	0.7 (0.2)	–	
5–8 Months	7	0.7 (0.2)	7	0.6 (0.2)	−0.29 (−0.59 to 0.02)	0.4–1.0 g, Exercise only

* The overall treatment difference is estimated based on outcome at 5–8 months, calculated using a linear model with 5–8 months as outcome timepoint, adjusted for treatment allocation, baseline outcome score and frailty status. No p values are included as study is not powered for between group comparisons.

eGFR, estimated glomerular filtration rate; EQ-5D, EuroQol Group 5-Dimension; VAS, Visual Analogue Scale.

At follow-up and after participants had completed the interventions (including protein supplements), the exercise only arm had slightly better dietary protein intake than the exercise and protein arm, but both groups still had protein intake below the recommended 1–1.2 g/kgBW/day.

#### Adverse events

All AEs were from participants in the exercise only group. There was one serious AE. A participant fell and fractured their wrist while stepping off the bus after going on a long walk with a walking group that they had joined as part of their planned ongoing walking activities (classified as probably related to the intervention). There were two AEs. One participant had a fall at home unrelated to the intervention and another twisted her ankle while standing up to do the exercises. Both participants continued to attend the exercise group. There was one AE during an eligibility assessment for the trial (patient became unwell) and was deemed too unwell to join the study. No deaths were reported during the study.

## Discussion

Our findings show that a definitive trial evaluating the effectiveness of mobility and strength training with and without protein supplements in prefrail/frail older people with low protein intake was not feasible as originally proposed. However, much has been learnt to inform the protocol for a definitive trial.

Recruitment was the biggest challenge, and we only recruited 20 out of a target of 50 despite screening 952 patients. Due to NHS waiting list pressures, study sites focused on screening patients on their clinical caseload or waiting lists. Patients were more unwell or immobile (including housebound) than anticipated and were either ineligible or did not want to take part. At one site, the caseload was older patients with complex mental health issues. One of the main reasons for non-participation was travel difficulties, which are often reported as a barrier to participation in physical activity or exercise programmes for older people.^33 34^ We actively identified potential participants for 7 months, but the majority of eligible participants were identified during the final 3 months when we implemented a new recruitment strategy which involved advertising in local venues used by older people to reduce the burden of travel. This strategy was more successful, but the need to gain ethical and site approvals meant we could not implement this change quickly. The timeframe available for the study meant that we could not extend recruitment to fully test this strategy. Recruiting older adults who are frail or likely to have sarcopenia to supplement studies has been shown to be challenging in previous studies (eg, Witham *et al* recruited 145 participants out of a target of 440.^35^ Another recent feasibility study of strength training and nutritional supplements recruiting older people with sarcopenia reported recruitment as one of the main barriers to feasibility;^36^ hence, the need to undertake this feasibility study. We could deliver exercise via home-based exercises which would eliminate a major barrier to recruitment (travel), but a recent review demonstrated the difficulty in providing adequate exercise dose and fidelity of interventions when exercises are undertaken as home exercises.^37^

We required participants to have low protein intake, which led to the exclusion of a substantial number of potential participants. Of the 87 potential participants who agreed to telephone screening (with 41 attending research clinics), 19 (22%) had sufficient dietary protein intake and were thus ineligible. This would need to be taken into consideration when planning a larger trial. We could make recruitment easier by removing this criteria, but this approach is consistent with clinical guidelines^6^ and studies where participants have had sufficient dietary protein intake show that additional protein does not improve outcomes.^38^

Intervention fidelity and adherence were generally satisfactory, although we would recommend some changes for a definitive trial. The physiotherapists successfully delivered the exercise groups according to the protocol. We had reasonable attendance among those who started the intervention, with the main reason for non-attendance being sickness. An area of uncertainty prior to the trial was whether frail older people may find attending 16 weeks of classes to be burdensome, but observations of the groups revealed that participants were engaged and found the exercise sessions enjoyable. Average attendance rates were the same as those reported for similar community-based exercise groups,^39^ therefore, reflecting real life. The participating physiotherapists did express some concerns over the longer-term provision of the exercise classes and travel was a barrier to people joining the trial. Therefore, we are also considering whether we could use existing community-based strengthening exercise classes for older people in a larger future trial to reduce travel for participants, to promote long-term engagement with exercise and for future sustainable implementation. Although only a small number of participants received the supplements, these were taken regularly and were well tolerated. The physiotherapists delivering the interventions were able to manage the provision of supplements well with the support of the study dietitian. The only issue we identified with the supplements was that participants did not like the strawberry flavour, so we would only offer vanilla in a future study.

In addition to recruitment, one concern for a future study is the withdrawal rate. We had three withdrawals and one participant who never attended (20% of recruits). Follow-up was high among those who attended, but these withdrawals/non-attenders resulted in lower follow-up than anticipated (70% for the proposed primary outcome). Ways to engage and support attendance will be considered further for a definitive trial including locally provided exercise groups as discussed above.

There are some limitations to this study. First, we aimed to increase participants’ daily protein intake up to 1.6 g/kgBW/day, but this was not easily achieved since some participants had very low protein intake to begin with and the maximum dose was two drinks due to the calcium and vitamin D content of the supplement. For a future study, we could use a different supplement without this limitation. However, we selected this particular protein supplement as it was formulated to build muscle in people with sarcopenia by using the synergistic benefit of whey, leucine and Vitamin D enrichment that is proposed to be the best way to improve muscle mass and strength in frail older people,^40^ and participants found the chosen supplement very acceptable, and it was well tolerated. Asking participants to take more than two drinks a day may reduce adherence. Most participants were on the maximum of two drinks per day, which provided an additional 40 g of protein per day. Despite not achieving the originally planned increase in intake, the intake achieved is more than other studies that provided quite modest increases in protein. For example, in the review by Labata-Lezaun, at least eight of the included studies only provided an additional 25 g of protein per day, which is potentially insufficient to stimulate muscle protein synthesis.^41^ Also, we did not use a placebo supplement, so there was the possibility that participants in the exercise-only arm would increase their protein intake through their diet in response to not receiving the supplements; this could affect the study findings. However, this was not the case, and the dietary protein intake of those randomised to exercise only remained below the daily recommendations.

This was a feasibility study, so it was not powered to detect differences in clinical outcomes, and we only recruited 20/50 participants. There were baseline imbalances; those in the exercise and protein arm were younger and more physically able than those in the exercise only arm. However, both groups had substantial mobility disability based on their 6MWT, which was well below data for healthy cohorts.^42^ We adjusted for baseline values in the comparison to account for these differences. The clinical outcome comparisons should be interpreted with caution given the small number of participants, but there was a trend towards greater improvements in those who were allocated to exercise and protein relative to exercise only.

A final limitation was that nearly all the participants were white, despite having sites in Birmingham and Bradford where the population is more ethnically diverse. The community recruitment plan focused on proximity to venues for the exercise classes rather than a strategy to include people from ethnic minorities. Therefore, the population was not representative of pre-frail or frail older people across the UK. For a definitive trial, we would develop a clear recruitment plan to include a more diverse population.

## Conclusions

The study was not feasible based on the original protocol. Recruitment was the biggest challenge. We established a more efficient route to recruitment (community advertising) which requires further refinement. Clinical outcomes consistently favoured the exercise and protein group, and although this should be interpreted cautiously, it suggests this question is worthy of further investigation. We will continue to work with stakeholders to develop the protocol for a definitive trial.

## Supplementary material

10.1136/bmjopen-2025-102411 online supplemental file 1

## Data Availability

Data are available upon reasonable request.
