# Supplementary material for: Mobility and strength training with and without protein supplements for pre-frail/frail older people with low protein intake: maximising mobility and strength training (MMoST) feasibility randomised controlled trial
Source: BMJ Open. 2026 Jan 7;16(1):e102411. doi: 10.1136/bmjopen-2025-102411 (PMC12781993; doi:10.1136/bmjopen-2025-102411)
Supplement: online supplemental file 1 [file bmjopen-16-1-s001.docx]

Supplementary Figure 1 Study Flow Chart

**Participant identification**

**Follow up:** 5-8 months

Face-to-face appointment with site research team (or home visit if participant unable to attend). Patient completes questionnaire and physical tests. If an in-person visit is not possible then a questionnaire will be posted to the participant.

2 x 24hr dietary assessments

Blood test (if no pre-existing blood test results within the last 1 month are available).

**Research Clinic Appointment**

Face-to-face appointment with site researcher for eligibility assessment

**Consent:** If eligible & willing to take part then participants provide written informed consent to undergo the full eligibility assessment and to take part in the study.

The assessment includes two 24-hour dietary assessments and a blood test to check renal function (if no pre-existing blood test results within the last 3 months are available).

**Eligibility assessment:** 1^st^ 24-hour dietary assessment completed, and blood test undertaken if required.

**Baseline data collection:** Patient completes questionnaire and physical tests.

**Telephone screening**

Research staff (site or central study team) call potential participant, initial screening undertaken, verbal consent taken for initial dietary assessment of protein and to access pre-existing blood results (within the last 3 months). Participants invited for a full eligibility check if they appear potentially eligible and willing.

.

**Interventions:** The participant attends for allocated treatment

**NHS rehabilitation services**

**(e.g. physiotherapy or falls clinics)**

**OPAL cohort study**

**Community advertisements**

**Excluded**

Did not meet eligibility (reason recorded)

Patient declined to participate (reason recorded if given)

**Final eligibility checks**

**Blood test results:** central study team access results received from blood test if taken at research clinic

**2^nd^ 24-hour dietary assessment:** Participant completes this at home or during phone call from central study dietitian.

**Randomisation**

**Mobility and Strength Training**

**+ Protein Supplements**

Mobility and Strength Training as described.

Additional individual appointment (30 minutes) to provide daily protein supplements tailored to the individual based on baseline dietary assessment.

Protein supplements taken for 24 weeks with short review at 2,4,8,12,16 weeks after group session

**Mobility and Strength Training**

Attends individual appointment (60 minutes) for assessment and prescription of individually tailored, progressive exercises.

16 weeks – weekly group session (60 minutes) supplemented with one session per week of home training.

8 weeks – independent home training twice per week with physiotherapy support.

Supplementary Table 1: Randomisations by site

| **Randomisation** | **Mar-23** | **Apr-23** | **May-23** | **Jun-23** | **Jul-23^1^** | **Aug-23** | **Sep-23^2^** | **Oct-23** | **Nov-23** | **Total** |
| --- | --- | --- | --- | --- | --- | --- | --- | --- | --- | --- |
| **Site 1 (opened 1 March 2023)** | 0 | 0 | 0 | 0 | 0 | 0 | 0 | **3** | **2** | **5** |
| **Site 2 (opened 30 March 2023)** | 0 | 0 | 0 | 0 | **3** | **2** | 0 | **2** | 0 | **7** |
| **Site 3 (opened 26 April 2023)** | - | 0 | 0 | 0 | 0 | **2** | **3** | **1** | 0 | **6** |
| **Site 4 (opened 20 June 2023)** | - | - | - | 0 | 0 | 0 | **2** | 0 | 0 | **2** |
| **Total** | **0** | **0** | **0** | **0** | **3** | **4** | **5** | **6** | **2** | **20** |

^1^ Amendment to recruitment introduced to include advertising in the community

^2^Identification of potentially eligible people stopped at the end of September and sites completed eligibility assessments and randomisation. Grey shaded area is active recruitment phase.

Supplementary Table 2: Protein supplement prescription (as randomised, n=10)

|  | **Mobility and strength training plus protein supplements (n=10)** |
| --- | --- |
| **Protein limited to 1.3 due to renal function (eGFR between 30-60ml/min/1.73m²)^1^** | 3 (30%) |
| **Baseline dose prescribed based on protein gap (drinks)^1^** |  |
| 8-17.9g (0.5 drinks) | 0 (0%) |
| 18-27.9g (1.0 drinks) | 2 (20%) |
| 28-37.9g (1.5 drinks) | 0 (0%) |
| >38g (2.0 drinks) | 8 (80%) |
| **Daily protein intake that would have been achieved with supplement (****g/kgBW/day)^1^** |  |
| 1.1 - 1.19 | 2 (20%) |
| 1.2 - 1.29 | 2 (20%) |
| 1.3 - 1.39 | 2 (20%) |
| 1.4 - 1.49 | 3 (30%) |
| 1.5 - 1.60 | 1 (10%) |

*^1^Summaries are n (%)*
