# Supplementary material for: Mobility and strength training with and without protein supplements for pre-frail/frail older people with low protein intake: maximising mobility and strength training (MMoST) feasibility randomised controlled trial
Source: BMJ Open. 2026 Jan 7;16(1):e102411. doi: 10.1136/bmjopen-2025-102411 (PMC12781993; doi:10.1136/bmjopen-2025-102411)
Supplement: Supplementary file 2 [file bmjopen-2025-102411.reviewer_comments.pdf]

PEER REVIEW HISTORY

BMJ Open publishes all reviews undertaken for accepted manuscripts. Reviewers are asked to complete a checklist review form and are provided with free text boxes to elaborate on their assessment. These free text comments are reproduced below.

ARTICLE DETAILS

Title (Provisional)

Mobility and strength training with and without protein supplements for pre-frail/frail older people with low protein intake: Maximising Mobility and Strength Training (MMoST) Feasibility Randomised Controlled Trial

Authors

Williamson, Esther; Biggin, Kavita; Morris , Alana; Marian, Ioana; Mwema, Christopher; Carver , Andrew; Lamb, Sarah Elizabeth

---

VERSION 1 - REVIEW

**Reviewer**	**1**
**Name**	**Beck, AM**
**Affiliation**	**Herlev Hospital, EATEN**
**Date**	**20-May-2025**
**COI**	**None**

The topic of the paper is very relevant, so is the choosing of performing a feasibility study before initiating a definite trial, the use of the stop-go criteria and the Patient and Public involvement. However, there still are some issues which need to be addressed. These are described below.

Abstract

The objective and the conclusion should align more closely to the objective and conclusion presented in the manuscript.

Methods

P6. Design.

It could be relevant to add that the study is reported according to the consort guidelines for pilot and feasibility studies, and eventually refers to the appendix with details about this

P6. Setting and P7. Participants.

These two sections should be aligned so it is easier to understand what is done where, and by whom – for example by repeating the three recruitment sites mentioned on page 6 again on page 7 (line 26, line 35 and line 47).

P7. Line 38 – it is difficult to understand with ''only one study site was willing to accept OPAL participants''? Please explain.

P10. Line 23. – please explain why there was a maximum dose of intake of the supplement - is it due to the Upper level of the two micronutrients or?

P10. Line 50. Please provide a reference for the KDIGO guideline

P11. Line 22. Please provide a reference for the use of the traffic light system.

P11. Line 30. What is meant by ''the viability' of data collection?

A suggestion is to define a specific cut-off – for example above 80% completeness - to determine whether the outcome assessment is feasible – including a reference to the chosen cut-off.

P11. Line 39. Please describe in more detail what is considered an adverse event.

Results

P12. L. 52. It could be relevant to also refer to table 4 here.

It is not clear how the PPI was involved in the decisions taken regarding the traffic-light colors of the different outcomes – this needs to be described.

The viability of data collection needs to be described.

Discussion

The discussion is quite long and not so easy to follow. If the journal allows a suggestion could be to structure the discussion the same way as the result section.

The discussion of feasibility needs to be based on references justifying the different stop and go criteria's - see also comment to table 4 below - not the least in relation to the ''red'' since this has a major impact on the future trial.

Again it would be nice to have a description on how the PPI was involved in this part of the study.

Tables and figures

Table 3. Please describe, respectively, the exercise and protein supplements taken diary mentioned here in the method section as well

To use the EQ5D5L approval must be obtained from the Europol organization - please state that such an approval has been obtained.

Table 4. Please provide some references to justify the different stops and go cut-offs/criteria's

Table 5. May not be necessary

Table 6. A suggestion is to use this table to also show completes of data collection.

Figure 1 does not comply with a traditional consort flow diagram – a suggestion is to use one of the examples used in the Consort paper regarding pilot and feasibility trials.

Supplementary Figure 1 Study flow chart. It seems to be the proposed flow of the study – if so, there should be no mentioning of number (N=25)

Supplementary table 1 and 2 may not be necessary, nor do the detailed protocol.

**Reviewer**	**2**
**Name**	**Prokopidis, Konstantinos**
**Affiliation**	**University of Liverpool**
**Date**	**11-Jun-2025**
**COI**	**None**

This is a well-written and important paper, particularly for researchers and clinicians working with older adults with frailty.

Some minor comments below:

Introduction --> I would add the sentence "Exercise including...associated with frailty (6,7)" towards the end of the paragraph to link it with the objectives of your study.

Methods --> Please add "UK" next to Birmingham in the "Study Participants" section.

- I am not seeing the protocol's code. I think it would be of value.

- In section "Protein supplements", please describe what type of 'fibres' or just mention "x g of dietary fibre"

- Could participants consume the protein supplements any time of the day or in conjuction with other meals? Please provide a description to make it more clear in your "Protein supplements" section.

- How did you ensure adherence of participants for at home exercise?

- Throughout your manuscript please pay attention on the first abbrevation of certain words/phrases. That would include 6-minute walk test or adverse events.

**Reviewer**	**3**
**Name**	**Kelson, Zoe**

**Affiliation**	**University of Exeter, Mathematics**
**Date**	**21-Aug-2025**
**COI**	**None**

This multi-centre feasibility randomised controlled trial aims to establish the feasibility of conducting a definitive trial to evaluate the effectiveness of mobility and strength training with or without protein supplements for pre-frail/frail older people with low protein intake.

Reviewer comments:

"All clinical outcomes showed a trend towards larger improvements in the exercise and protein arm but these were not statistically significant" [Abstract]

Can the Abstract please summarise statistical methods and report statistical outcomes alongside stated study findings?

"A multi-centre, parallel, 2-group, feasibility randomised (1:1) controlled trial (See Figure 1). "

A rigorous study design has been proposed.

Thanks for providing a copy of the study protocol.

"Four NHS community trust physiotherapy departments. We recruited via clinical caseloads, an existing cohort study and community advertising" [Abstract]

and

"We identified potential participants via: 1. Four NHS community trusts in England. 2. An existing primary care-based cohort study. 3. Community advertising (introduced in the later phase of recruitment)."

and

"Due to poor recruitment, we implemented a new community-based recruitment strategy in the later stages of recruitment. "

Can the authors please further comment on whether the included cohort can be considered to be demographically and characteristically representative?

"The target sample size was 50 based on a recent simulation study of sample size requirements in pilot RCTs which recommends at least 50 subjects are needed to calculate key design parameters and establish the definitive study sample size "

The basis for the sample size has been communicated.

"The analyses were conducted according to a pre-specified statistical analysis plan"

Can the authors please provide a copy of this statistical analysis plan (SAP), or are they referring to the statistical analysis section within the study protocol?

"Descriptive statistics of the feasibility outcomes are reported (recruitment rates, reasons for ineligibility, intervention fidelity and adherence, retention rates). Participant baseline demographic and clinical characteristics for each randomised group and overall are described. "

and

"Completed forms had very little missing data"

and

"We will explore missingness for each outcome to inform final selection of outcomes for the definitive RCT" [protocol]

and

"Missing data will be described with reasons given where available" [protocol]

Can the authors please comment on whether missing data can be considered to be missing at random?

"Participant reported outcomes are summarised as a mean (standard deviations) or median (interquartile ranges) and frequency with percentages for continuous and categorical outcomes, respectively"

Can the authors please clarify how normality was assessed for the choice of parametric or non-parametric statistical descriptors?

"The mean between group differences at follow-up were estimated for each continuous outcome using linear regression models adjusted for baseline outcome score and presented with associated 95% Confidence Intervals (CI). These was exploratory analyses to test for a possible signal of treatment effect in the data. No p-values were included as the study was not powered for between group comparisons."

Can p-values please be reported, as exploratory only?

"Randomisation was 1:1, stratified by frailty (pre-frail/frail) using variable block sizes of 2 and 4 in a 1:1 ratio. A decision was made to allocate people in the same household to the same treatment based on the participant randomised first due to the risk of contamination. "

Can stratification variables please be adjusted for in all analyses?

Can clustering by household please be adjusted for in the sample size assessment and analytical methods?

"All participants as randomised who were not withdrawn from the study before their treatment started, and provided data, were included in the analyses (modified intent-to-treat (ITT))"

Can the authors please consider undertaking a Per Protocol (PP) sensitivity analysis?

"In total, 20 participants were randomised (10 in each arm). "

The challenges in recruitment have been transparently discussed throughout the article

"Outcome assessors were blinded but, as this was a pragmatic study and we did not use a placebo protein supplement, participants could not be blinded to their treatment allocation. • Recruitment did not proceed as anticipated, and changes were made to the protocol which took time to implement leaving insufficient time for recruitment. • Including participants with low protein intake added a level of complexity to this study but it was an important inclusion criteria as international guidelines recommend the use of protein supplements for older people when dietary intake is insufficient. • As it was a feasibility study, we cannot draw any conclusions about the effectiveness of the interventions. " [Strengths and Limitations of this study]

and

"However, there were several limitations. This was a feasibility study, so it was not powered to detect differences in clinical outcomes and we only recruited 20/50 participants. There were baseline imbalances; those in the exercise and protein arm were younger and more physically able than those in the exercise only arm. However, both groups had substantial mobility disability based on their 6MWT which was well below data for healthy cohorts (33). We adjusted for baseline values in the comparison to account for these differences. The clinical outcome comparisons should be interpreted with caution given the small number of participants, but there was a trend towards greater improvements in those who were allocated to exercise and protein relative to exercise only. Another limitation was that nearly all the participants were white, despite having sites in Birmingham and Bradford where the population is more ethnically diverse. The community recruitment plan focused on proximity to venues for the exercise classes rather than a strategy to include people from ethnic minorities. Therefore, the population was not representative of pre-frail or frail older people across the UK. For a definitive trial, we would develop a clear recruitment plan to include a more diverse population. "

The authors provide a discussion on the study limitations.

Thanks for providing a copy of the CONSORT checklist.

VERSION 1 - AUTHOR RESPONSE

Reviewer: 1

Abstract
The objective and the conclusion should align more closely to the objective and conclusion presented in the manuscript.

Response: We have added the second objective (finalise outcomes) to the abstract as suggested and revised the conclusion to better reflect the conclusion in the main manuscript.

Methods

P6. Design.

It could be relevant to add that the study is reported according to the consort guidelines for pilot and feasibility studies, and eventually refers to the appendix with details about this.

Response: We have added that we have reported according to the CONSORT guidelines for pilot and feasibility studies to the text with the reference.

P6. Setting and P7. Participants.

These two sections should be aligned so it is easier to understand what is done where, and by whom – for example by repeating the three recruitment sites mentioned on page 6 again on page 7 (line 26, line 35 and line 47).

Response: We have added headings to make this easier to follow.

P7. Line 38 – it is difficult to understand with ‘’only one study site was willing to accept OPAL participants’’? Please explain.

Response: Two sites were suitably located to recruit via the OPAL cohort study, but one site would not accept OPAL participants as due to waiting list pressures they only wanted to recruit from their existing caseload. This has been added to the text.

P10. Line 23. – please explain why there was a maximum dose of intake of the supplement - is it due to the Upper level of the two micronutrients or?

Response: Yes, the maximum does was two servings due to the vitamin D and calcium content. Greater than two would have exceeded the daily recommended limits. This has been clarified in the text.

P10. Line 50. Please provide a reference for the KDIGO guideline

Response: The reference has been added.

P11. Line 22. Please provide a reference for the use of the traffic light system.

Response: The reference has been added.

P11. Line 30. What is meant by ‘’the viability’ of data collection?

A suggestion is to define a specific cut-off – for example above 80% completeness - to

determine whether the outcome assessment is feasible – including a reference to the chosen cut-off.

Response: Viability refers to whether the procedures for collecting data worked successfully including consideration of the time taken to undertake data collection and burden on participants, any assistance needed by participants, if the researcher was correctly following the protocol when undertaking data collection and the completeness of data collected. The purpose was for refining data collection methods in a future definitive trial. This has been clarified in the text. We did not set a cut point for completeness, and we feel it would not be appropriate to set this retrospectively, but we have now reported data completeness in the results.

P11. Line 39. Please describe in more detail what is considered an adverse event.

Response: We have added more information to the text.

Results

P12. L. 52. It could be relevant to also refer to table 4 here.

Response: Table 4 is sign posted as suggested.

It is not clear how the PPI was involved in the decisions taken regarding the traffic-light colours of the different outcomes – this needs to be described.

Response: More information has been added to the PPI section.

The viability of data collection needs to be described.

Response: We have added information about data completeness to the results.

Discussion

The discussion is quite long and not so easy to follow. If the journal allows a suggestion could be to structure the discussion the same way as the result section. The discussion of feasibility needs to be based on references justifying the different stop and go criteria's - see also comment to table 4 below - not the least in relation to the ''red''

since this has a major impact on the future trial. Again it would be nice to have a description on how the PPI was involved in this part of the study.

Response: We have restructured the discussion based on the stop go criteria: recruitment, intervention fidelity and adherence and then retention as suggested and I hope it is easier to follow. Also, I have removed some text not relevant to these sections. The main reference that influenced selection of stop go criteria is included in the methods section and we have also expanded the section on PPI.

Tables and figures
Table 3. Please describe, respectively, the exercise and protein supplements taken daily mentioned here in the method section as well

Response: Some more information regarding the exercises and protein supplements has been added to Table 3.

To use the EQ5D5L approval must be obtained from the Europol organization - please state that such an approval has been obtained.

Response: The we had approval to use this has been added as a footnote to Table 2.

Table 4. Please provide some references to justify the different stops and go cut-offs/criteria's

Response: More details about how stop-go criteria has been added to the methods section including a reference.

Table 6. A suggestion is to use this table to also show completes of data collection.

Response: there was no missing data at baseline. This has been added to the text so it is clear.

Figure 1 does not comply with a traditional consort flow diagram – a suggestion is to use one of the examples used in the Consort paper regarding pilot and feasibility trials.

Response: We included additional information to the CONSORT flow diagram to provide detail about the different stages of eligibility screening and assessment. We felt this was important to understand where/when potential participants were excluded to inform future design of a definitive trial. We had added two additional steps to the diagram to make it better aligned with the CONSORT flow diagram as you suggest. We additionally clarified the reasons for exclusion during screening and reasons for not receiving the allocated treatment or loss-to-follow-up post randomisation.

Supplementary Figure 1 Study flow chart. It seems to be the proposed flow of the study – if so, there should be no mentioning of number (N=25)

Response: numbers have been removed.

Table 5. May not be necessary
Supplementary table 1 and 2 may not be necessary, nor so the detailed protocol.

Response: These have been retained unless the Editor wants us to remove them.

<u>Reviewer: 2</u>

Some minor comments below:
Introduction --> I would add the sentence "Exercise including...associated with frailty (6,7)" towards the end of the paragraph to link it with the objectives of your study.

Response: We have made this change as suggested.

Methods --> Please add "UK" next to Birmingham in the "Study Participants" section.

Response: This section was edited in response to another reviewer comments and the name of the site has been removed. In the section titled "Setting" we specify that the four study sites are in England.

- I am not seeing the protocol's code. I think it would be of value.

Response: The trial registration number is provided as part of the abstract (ISRCTN30405954) and the reference for the protocol has now been included at the start of the methods section.

- In section "Protein supplements", please describe what type of 'fibres' or just mention "x g of dietary fibre"

Response: The amount of fibre has been added (1.3g of dietary fibre)

- Could participants consume the protein supplements any time of the day or in conjuction with other meals? Please provide a description to make it more clear in your "Protein supplements" section.

Response: This detail has been added to the text.

- How did you ensure adherence of participants for at home exercise?

Response: Behavioural change strategies were embedded into the programme to encourage adherence with the home exercises. These are outlined in the protocol

paper which is referenced in the current paper. These included exercise planning, goal setting and using an exercise diary. The strategies are listed in Table 3.

- Throughout your manuscript please pay attention on the first abbreviation of certain words/phrases. That would include 6-minute walk test or adverse events.

Response: We have checked these and corrected as needed.

Reviewer: 3
Prof. Zoe Kelson, University of Exeter
Comments to the Author:
Reviewer comments:

"All clinical outcomes showed a trend towards larger improvements in the exercise and protein arm but these were not statistically significant" [Abstract]
Can the Abstract please summarise statistical methods and report statistical outcomes alongside stated study findings?

Response: We have added further information to the abstract as requested.

0.93 (-2.70,4.56)
41.92 (-39.05,122.89)
"Four NHS community trust physiotherapy departments. We recruited via clinical caseloads, an existing cohort study and community advertising" [Abstract]
and
"We identified potential participants via: 1. Four NHS community trusts in England. 2. An existing primary care-based cohort study. 3. Community advertising (introduced in the later phase of recruitment)."
and
"Due to poor recruitment, we implemented a new community-based recruitment strategy in the later stages of recruitment. "
Can the authors please further comment on whether the included cohort can be considered to be demographically and characteristically representative?

Response: Yes, the OPAL cohort is broadly representative of the general population of people of the same age, and we had added this to the text with a reference.

"The analyses were conducted according to a pre-specified statistical analysis plan"
Can the authors please provide a copy of this statistical analysis plan (SAP), or are they referring to the statistical analysis section within the study protocol?

Response: The analysis plan followed was as described in the study protocol. We considered that a separate SAP would not add much value, given that

pilot/feasibility studies often use descriptive, non-inferential analysis approaches. We have (in house) prepared dummy tables ahead of the analysis stage to help guide the pre-specified analysis.

"Descriptive statistics of the feasibility outcomes are reported (recruitment rates, reasons for ineligibility, intervention fidelity and adherence, retention rates). Participant baseline demographic and clinical characteristics for each randomised group and overall are described. "
and
"Completed forms had very little missing data"
and
"We will explore missingness for each outcome to inform final selection of outcomes for the definitive RCT" [protocol]
and
"Missing data will be described with reasons given where available" [protocol]

Can the authors please comment on whether missing data can be considered to be missing at random?

Response: We have now reported the amount of missing data in the paper. There was no missing data at baseline. Among participants who attended their follow up appointment, there was also no missing data at follow up in the participant completed questionnaires or related to the physical tests. The only missing data at follow up was for dietary assessment (n=1) and three missing blood test results (n=1 in usual care and n=2 in intervention group). Given the small number of missing items and a small study sample, it is difficult to make reasonable comparisons or correlations.

"Participant reported outcomes are summarised as a mean (standard deviations) or median (interquartile ranges) and frequency with percentages for continuous and categorical outcomes, respectively"
Can the authors please clarify how normality was assessed for the choice of parametric or non-parametric statistical descriptors?

Response: We used visual inspection to examine the shape of each continuous variable distribution. However, given our small sample size (≤10 per group) we chose to report medians with IQR for most continuous values as a more robust approach. In the analysis of clinical outcomes and participant reported outcomes, we presented the means to accompany the between-group mean difference and aid with interpretation. We have now added clarification in the Statistical methods section.

"The mean between group differences at follow-up were estimated for each continuous

outcome using linear regression models adjusted for baseline outcome score and presented with associated 95% Confidence Intervals (CI). These was exploratory analyses to test for a possible signal of treatment effect in the data. No p-values were included as the study was not powered for between group comparisons."
Can p-values please be reported, as exploratory only?

Response: In our pre-specified analysis plan we said "For this feasibility study statistical significance will not be assessed. Comparative results will be presented using 95% Confidence intervals (CI) with no statistical testing. These are expected to be wide due to the small sample size and care should be taken with any interpretation." In line with this and the CONSORT 2010 Extension for Pilot and Feasibility Trials (Eldridge et al., 2016) we feel presenting p-values to accompany comparisons would be inappropriate. We are instead placing emphasis on estimating parameters (e.g., recruitment rate, adherence, data completeness), rather than detecting significant differences in outcomes.

"Randomisation was 1:1, stratified by frailty (pre-frail/frail) using variable block sizes of 2 and 4 in a 1:1 ratio. A decision was made to allocate people in the same household to the same treatment based on the participant randomised first due to the risk of contamination. "
Can stratification variables please be adjusted for in all analyses?
Can clustering by household please be adjusted for in the sample size assessment and analytical methods?

Response: Thank you for this comment. Our original analyses models included the baseline outcome score as well as frailty status. We have now additionally clarified this in the text and table footnotes.

The trial was not designed to allow for more than a single participant to be recruited from the same household and therefore the sample size did not account for such clustering. It was noted during recruitment that two participants belonged to the same occupancy and therefore this pragmatic decision to provide the same treatment was made. We acknowledge that for a full size trial, the sample size and analysis methods would need to be adjusted to account for such clustering effect.

"All participants as randomised who were not withdrawn from the study before their treatment started, and provided data, were included in the analyses (modified intent-to-treat (ITT))"
Can the authors please consider undertaking a Per Protocol (PP) sensitivity analysis?

Response: Thank you for these suggestions. The adjustments suggested were not planned in the original statistical analysis plan and we are not planning any further

analyses as the funded study period is over. It is unlikely, considering the small sample size and exploratory nature of the analyses, that we could draw any firmer conclusions from additional analyses. However, we will take these recommendations into consideration when planning the definitive trial.

VERSION 2 - REVIEW

**Reviewer**	**1**
**Name**	**Beck, AM**
**Affiliation**	**Herlev Hospital, EATEN**
**Date**	**13-Oct-2025**
**COI**	

The authors have replied satisfactorily to all my comments except the one regarding the red, amber and green criteria described in table 4

Just stating ''Stop-go criteria were decided by the study team based on published recommendations (24), our experience of delivering large clinical trials and our understanding of exercise and protein supplement dosing and through discussion with our Patient and Public Involvement group, where ref 24 is a general reference in relation to the stop-go criteria is not sufficient. Hence there needs to be justification for each of the selected cut-offs presented in table 4, based on the available literature regarding each item (1-7)

VERSION 2 - AUTHOR RESPONSE

Reviewer: 1
Dr. AM Beck, Herlev Hospital
Comments to the Author:

Just stating ''Stop-go criteria were decided by the study team based on published recommendations (24), our experience of delivering large clinical trials and our understanding of exercise and protein supplement dosing and through discussion with our Patient and Public Involvement group, where ref 24 is a general reference in relation to the stop-go criteria is not sufficient. Hence there needs to be justification for each of the selected cut-offs presented in table 4, based on the available literature regarding each item (1-7)

Response:
I have added more details about setting the stop go-criteria focusing on the criteria set for green light. I hope this is sufficient to address the reviewer comment above.
